# Bone Marrow Niches of Hematopoietic Stem and Progenitor Cells

**DOI:** 10.3390/ijms23084462

**Published:** 2022-04-18

**Authors:** Oleg Kandarakov, Alexander Belyavsky, Ekaterina Semenova

**Affiliations:** Engelhardt Institute of Molecular Biology, Russian Academy of Sciences, 119991 Moscow, Russia; oleg.kandarakov@europe.com (O.K.); katisemenova@hotmail.com (E.S.)

**Keywords:** hematopoiesis, hematopoietic stem cells, niches, bone marrow, hypoxia, mesenchymal stromal cells, metabolism, aging, leukemogenesis, 3D modeling

## Abstract

The mammalian hematopoietic system is remarkably efficient in meeting an organism’s vital needs, yet is highly sensitive and exquisitely regulated. Much of the organismal control over hematopoiesis comes from the regulation of hematopoietic stem cells (HSCs) by specific microenvironments called niches in bone marrow (BM), where HSCs reside. The experimental studies of the last two decades using the most sophisticated and advanced techniques have provided important data on the identity of the niche cells controlling HSCs functions and some mechanisms underlying niche-HSC interactions. In this review we discuss various aspects of organization and functioning of the HSC cell niche in bone marrow. In particular, we review the anatomy of BM niches, various cell types composing the niche, niches for more differentiated cells, metabolism of HSCs in relation to the niche, niche aging, leukemic transformation of the niche, and the current state of HSC niche modeling in vitro.

## 1. Introduction

The hematopoietic system, with its ability to produce an estimated half a trillion new cells per day in humans [1], is arguably the most “prolific” system in a body. Yet this formidable number is generated thanks to only a wmqll tiny quantity—about one million [2], extremely rare HSCs residing in BM [3]. HSCs produce all mature blood and immune cells of the body, with the exception of a few special cell subpopulations such as tissue-resident macrophages and innate-like B and T lymphocytes [4,5,6].

The hematopoietic system, in addition to its utmost efficiency in mature cell production, is also highly adaptable yet very tightly regulated. HSCs are extremely quiescent cells that enter cell cycle quite rarely [7,8]. While in the state of quiescence, HSCs, however, remain on high alert, and in situations of dire organismal need such as major blood loss or microbial invasion, may enter the cell cycle to replenish exhausted progenitors and boost production of effector cells [9,10].

Due to quiescence of the majority of HSCs, homeostatic hematopoiesis is mostly performed through expansion of more differentiated progeny cells that are biased or committed to certain hematopoietic lineages [11,12]. As these cells have limited self-renewal ability and are not long-lived [13], the normal hematopoiesis appears as a succession of a large number of different clones [14]. HSCs themselves have an extensive self-renewal potential that is substantially higher than that of the immediate progeny but is not limitless as it declines with hematological stresses or age [15,16].

The purest and seemingly homogeneous HSC samples that can be obtained by cell sorting are nevertheless fairly heterogeneous, as evidenced by recent results employing lineage tracing and single cell RNA sequencing approaches. These sophisticated techniques revealed the existence in vivo of HSCs biased towards certain lineages, as well as non-classical differentiation routes bypassing multipotent progenitors and directly generating lineage-restricted progenitors [16,17]. In particular, these results indicate the existence of megakaryocyte-biased HSCs and the megakaryocyte differentiation pathway bypassing the stage of common myeloid precursors [18,19]. Thus, although the view of hematopoiesis as a hierarchically organized ensemble of developmentally related cell populations remains valid, the current models of hematopoiesis [20] allow for a substantially higher flexibility in cell fate decisions, as was previously considered possible [21].

## 2. Anatomy of the BM Niche

In 1978, Schofield, analyzing outcomes of hematopoietic transplantation experiments, proposed the concept of niche as a defined anatomical location that is required for HSCs in vivo to exist and fulfill their functions [22]. He further postulated that the niche has an instructive role in vital decisions of HCSs and their self-renewal is only possible within the niche, whereas HSCs leaving the niche embark on their differentiation journey. The general validity of Schofield’s concept was later confirmed first in Drosophila by identification of stem cell niches in gonads [23], and later in other cell systems.

HSCs during the adult mammalian life reside in BM, which thus serves as a macro-niche for HSCs. Bone cavities contain trabecula, a type of spongy bone tissue that is actively remodeled and in long bones is located in the epiphysis and metaphysis. The BM itself is located between the trabeculae and consists of a loose stroma permeated with vessels, stromal cells and various hematopoietic cells. The periosteal and feeding arteries supplying the BM pass into the bone marrow cavity of the long bones through the nutrient channel, giving rise to smaller arterioles [24,25]. The arterioles then connect to the vascular sinusoids, peculiar fenestrated capillaries with a wide lumen, which in turn connect to a longitudinal central venous sinus flowing into veins exiting through the nutrient channel. The arterioles are in close contact with sympathetic nerve fibers [26] and are also covered by perivascular mesenchymal cells and non-myelinating Schwann cells.

Although BM can be considered a macro-niche for HSCs in mammalians, this notion is lacking the necessary cellular detalization, and the need for detailed characterization of cellular and molecular mechanisms controlling HSC function spawned a race for identification of bona fide HSC niches in BM. Osteoblast located in the endosteal region were the first proposed candidate for the cellular component of the HSC niche as reported by two groups in 2003 [27,28]. In the study published next year, selective elimination of osteoblasts resulted in the decrease of HSC numbers with simultaneous activation of extramedullary hematopoiesis in the spleen and liver [29]. These works were, however, indirect and did not provide solid proof of physical contacts or close association between osteoblasts and HSCs. Subsequently, the attention of research teams was attracted to other cellular components of BM as potential components of the niche.

Major technical improvements, in particular identification of SLAM receptors as appropriate HSCs markers [30], development of mouse strains with HSCs or potential niche components marked by fluorescent proteins, and intravital microscopy—significantly advanced understanding of HSC niches in BM. However, despite intensive ongoing efforts, the nature of the hematopoietic niche remains controversial.

HSCs in the BM were shown to be located in hypoxia regions, with the lowest O_2_ levels in deeper peri-sinusoidal regions, while the endosteal regions are less hypoxic [31]. Some studies indicate that HSCs are located proximal to arterioles in the endosteal area [32,33], whereas other reports demonstrate that HSCs are located in the vicinity of sinusoids [34]. Yet another work demonstrated the most quiescent long-term repopulating subset of HSCs to reside close to both sinusoidal blood vessels and the endosteal surface [35]. A careful study revealed later that the frequency of apparent association HSCs with candidate niches correlated with the abundance of those niches in the BM. Importantly, the distribution of HSCs relative to these niches was not different from distribution of computationally generated dots randomly placed throughout the BM volume [36]. This work demonstrates therefore the absence of a preferential association of adult HSCs with anatomical locations in BM proposed as specific HSC niches. These unexpected findings suggest that misinterpretation of data on HSC-niche co-localization might be a source of discrepancies between the results obtained by different groups and demonstrate that appropriate controls are of primary importance for correct analysis of such extremely complex biological processes as hematopoiesis.

The discordant concepts placing HSC locations near arterioles or sinusoids may possibly be reconciled by studies suggesting that myeloid-biased HSCs may be localized near megakaryocytes in the vicinity of sinusoids, whereas lymphoid-biased HSCs seem to be located near arterioles [37,38]. If this concept is correct, it still remains to be elucidated whether the unbiased HSCs have a preference to specific anatomical locations in BM. It is also unclear whether the already pre-formed lineage-biased HSCs prefer the above-mentioned alternative locations, or if, vice versa, these locations induce lineage preference in the initially unbiased HSCs.

In general, the above studies demonstrate that there are a large number of functionally and anatomically similar locations in BM that HSCs may associate with, which implies that HSC niches are abundant and not a limiting factor in vivo. In accordance with this, transplantation of large quantities of HSCs into non-conditional hosts leads to their long-term engraftment while not replacing the recipient’s HSCs [39].

**Figure 1:**
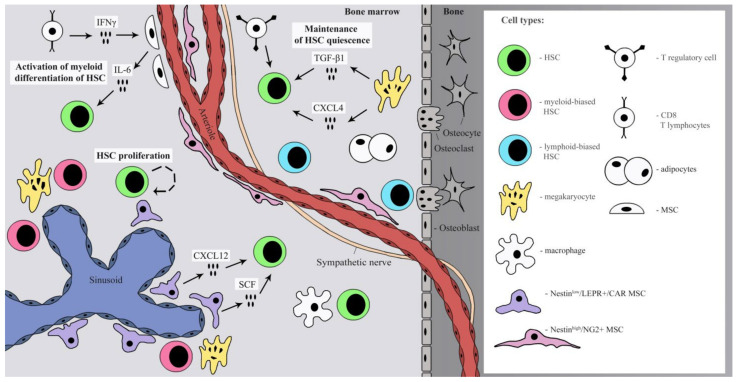
Cellular and selected molecular components of the HSC niche in BM. The reader is referred to the Section 3 for more detailed information on properties and roles of specific niche cell types.

A general overview of BM niche anatomy is provided in Figure 1.

## 3. Cell Components of the Niche

### 3.1. Mesenchymal Stem/Stromal Cells

Mesenchymal stem/stromal cells (MSCs) are a group of cells with multipotent capabilities which play an important role in tissue repair. These cells are characterized by a set of positive (CD73, CD90, CD105) and negative (CD14, CD19, CD34, CD45, HLA-DR) markers, the ability to differentiate into other cell types, such as osteoblasts, chondrocytes, adipocytes, and the secretion of various growth factors, cytokines and chemokines [40,41]. Substances secreted by MSCs play a key role in immunomodulation, as well as cell migration, proliferation, and differentiation. MSCs in BM are a rare population of cells (~0.001–0.01% of the total number of nuclear cells in BM aspirates), located perivascularly, directly on blood vessel surfaces along with sympathetic nerves [42], and play a prominent role in HSCs support [43,44,45]. Using different marker characteristics of MSCs (nestin (Nes), SCF (Stem Cell Factor), CXCL12, NG2, leptin receptor (LEPR)) and genetically modified mouse models, it was possible to distinguish different MSC subpopulations [27,46,47]. According to Kunisaki et al., MSCs in the periarterial and perisinusoidal niches differ in phenotypes and transcriptional profiles: Nes-GFP^bright^ and NG2^+^ MSCs were observed in the periarterial niche, while Nes-GFP^dim^ cells, LEPR^+^ and CXCL12^high^ MSCs are located in the perisinusoidal niche [32].

The regulatory role of BM MSCs towards HSCs was demonstrated by experiments with genetically modified mice. In particular, deletion of CXCL12 or SCF from all MSCs led to the depletion of the HSC population [28]. The MSCs populations in the periarterial and perisinusoidal niches have different effects on the preservation of HSC functionality: deletion of CXCL12 in the endosteal/periarterial niche NG2^+^ MSCs negatively affects the HSC population and leads to a redistribution of the remaining HSCs in BM away from arterioles, while deletion of CXCL12 in sinusoidal LEPR^+^ cells have no effect on HSC population. Results of Asada et al., however, indicate the importance of LEPR^+^ perisinusoidal cells rather than periarteriolar niche NG2^+^ cells as the main source of SCF needed to maintain HSC in the bone marrow [33]. The role of periarteriolar stromal cells in the resting state of HSCs was confirmed by a significant change in the distance between HSCs and arterioles after recovery from myelosuppression, polyinosin:polycytidylic acid (pIpC) treatment, or in Pml knockout mice, all of which led to excessive proliferation of HSCs, HSCs population exhaustion, and migration from arterioles [32].

It is interesting to note that the widely represented in the perisinusoidal niche MSC population with high expression of CXCL12, SCF, and LEPR (CAR cells) has a high expression of adipogenic and osteogenic factors, such as PPARy, Runx, and the ability to differentiate into osteoblasts and adipocytes [48,49]. Short-term ablation of CAR cells in mice led to a decrease in the HSC population in the niche [50], which was associated with reduced production of CXCL12 and SCF.

Nes^+^ MSCs are in close proximity to Schwann cells and the sympathetic nervous system involved in mobilization of HSCs. Removal of MSCs caused depletion of the HSC population in BM and increased their numbers in the spleen [45,51]. Co-cultivation of MSCs with HSCs increased survival and expansion of the latter [52].

MSCs secrete CXCL12 and a number of other factors such as SCF, VCAM-1, Angiopoietin-1, IL-6, IL-11, TPO (thrombopoietin), Flt-3 ligand, G-CSF, GM-CSF, M-CSF, and BMP4, which affect the HSC population and hematopoiesis in general [46,53,54]. The importance of SCF from MSC was confirmed by experiments on LepR-Cre mice [46]. The interaction of HSC and MSC is very multifaceted and is associated with the transmission of Wnt and Notch signals, the balance between which ensures self-renewal and maintenance of HSC. A member of another signaling pathway, BMP4, affects HSC during embryonic development, whereas high concentrations of this protein support the proliferation of HSCs from umbilical cord blood [55,56]. Conditional inactivation of the BMP4 receptor leads to an increase in HSC population in BM [27]. BMP4 can act directly on HSCs or through mediators like Shh (Sonic hedgehog). Shh in turn induces cytokine-dependent proliferation of HSCs [57]. Nes^+^ MSCs are involved in niche regulation via the beta-adrenergic receptor, which binds norepinephrine or adrenaline and affects physiological traffic of HSCs by modulating CXCL12 and SCF levels [58]. Of note, human CD45^−^CD105^+^CD146^+^ MSCs were able to create ectopic BM niche after subcutaneous or subrenal transplantation [59,60].

### 3.2. Endothelium

The endothelium in the niche is in direct contact with HSCs [30,47] and is represented by arteriolar and sinusoidal endothelial cells (ECs) [61]. These two cell groups differ in their functions: both groups produce SCF necessary for the survival of HSCs, although the sinusoidal ECs (SECs) synthesize only a small part of this factor [47,62]. At the same time, SECs produce large quantities of CXCL12 and E-selectin [63]. Arteriolar ECs (AECs), together with reticular cells, synthesize the extracellular matrix protein Del-1, which, through interactions with β3 integrin on HSCs, stimulates their proliferation and expansion, as well as differentiation towards myeloid lineage [64,65].

HSC self-renewal is stimulated by Notch ligands, which are synthesized by ECs [66]. In particular, Jagged-1 is an EC-produced Notch ligand that contributes to the maintenance of HSCs, since conditional deletion of Jagged-1 in ECs results in exhaustion of the HSC pool and severe decline of hematopoiesis [67]. Moreover, Jagged-1 participates in regeneration of hematopoiesis following myeloablation [66].

It should be noted that SECs are required for regeneratory hematopoiesis since lethal irradiation of BM produces their severe degeneration, while the regeneration of SECs is dependent on VEGFR2. Blocking SECs regeneration by neutralizing VE-cadherin or VEGFR2 antibodies prevents hematopoietic reconstitution as well [66,68]. A small subpopulation of Apln^+^ ECs also plays an important role in restoring the integrity of the niche [69]. The absence of these cells or deletion of the SCF and Vegfr2 genes disturbs vascular regeneration and maintenance of the HSC population following BM irradiation and HSC transplantation. This cell subset thus plays important role in the restoration of the vascular network [69].

While regeneration of SECs is critical for the restoration of HSC functions, SCF secreted by AECs also contributes to the regeneration of the HSC pool after irradiation [62,68]. EGF and some other factors secreted by Tie2^+^ ECs increase the survival and recovery of the HSC population after irradiation [70]. The importance of ECs is also demonstrated by the finding that erythromyeloid precursors can differentiate, if required, into ECs necessary for the restoration of the blood vessel network [71]. Co-cultivation of ECs with CD34^+^ hematopoietic progenitors contribute to an expansion of the latter [72]. Co-transplantation of endothelial progenitor cells with HSCs promotes endothelial cell recovery, as well as hematological and immune reconstitution [73].

Importantly, the Klf6 transcription factor expressed in ECs was shown to modulate HSC lodgment and expansion of HSCs in zebrafish via chemokine Ccl25b. Moreover, its mammalian ortholog Ccl21 was able to expand hematopoietic progenitors in the ex vivo system [74]. Of note, the loss of the vascular-endothelial-expressed Notch ligand DLL4 distorts bone marrow hematopoiesis towards significant transcriptional reprogramming and myeloid priming of HSC [75].

### 3.3. Osteoblasts

This cell type was the first one proposed to be a part of the hematopoietic niches [27,28,29]. In particular, long-term HSCs were found to be in contact with N-cadherin-positive (N-cad^+^) osteoblastic precursors lining the inner bone surface and supporting HSCs through, presumably, the BMP signaling pathway [27]. Ablation of N-cad^+^ osteoblasts caused irreversible changes in the function of HSC during homeostasis and regeneration [29]. N-cad^+^ osteoblasts were also suggested to control HSC state and functions via non-canonical Wnt signaling [76,77]. Despite this evidence, a number of subsequent studies downplayed a possible role of endosteal cells in the maintenance of HSCs. In particular, conditional deletion of important HSCs regulators CXCL12 or SCF in osteoblasts did not significantly affect HSCs [47,78].

However, recent study revived an interest to endosteal niches [79] by demonstrating that dormant HSCs that are resistant to myeloablation (reserve HSCs) were found to be in contact with N-cad^+^ mesenchymal progenitors in the endosteal region [80]. Furthermore, ablation of N-cad^+^ niche cells or inactivation of the SCF gene in them negatively affected reserve HSC maintenance during homeostasis and regeneration. In a recent in vitro co-culture study, adherence to osteoblasts favored self-renewal of HSCs [81].

In the context of possible involvement of endosteal cells in HSC support, it should be mentioned that osteoblasts synthesize many factors affecting HSC, such as TPO, angiopoietin-1 and osteopontin, which prevent an increase in the HSC population [82,83,84]. It has been shown that when osteoblasts are activated by parathyroid hormone, they produce high levels of the Notch ligand Jagged-1, expanding the HSC pool [28]. Osteoblasts were also reported to affect the state of silent HSCs through the signaling pathways Tie2/angiopoietin-1 and TPO/Mpl, contributing to the interaction of HSCs with niche components that promote quiescence and self-renewal of these cells [53,85].

Interestingly, osteoblasts affect not only HSCs, but also more differentiated descendants. In particular, selective elimination of osteoblasts negatively affects the pool of early lymphoid precursors, without at the same time affecting the HSCs themselves or the precursors of the myeloid line [86].

### 3.4. Megakaryocytes

Megakaryocytes (MKs) reside predominantly in BM and, through production of platelets, are a prominent regulator of hemostasis and vascular integrity, with key roles in thrombosis and inflammatory responses. However, MK role is not limited to platelet production. As a result of sophisticated experimental studies of recent years, MKs also emerged as key regulators of HSCs in BM. Bruns et al., demonstrated that megakaryocytes regulate HSC quiescence [87]. They found nearly 30% of HSCs in BM localized close to MKs, and selective depletion of MKs led to HSC expansion as a result of their quiescence loss. MKs were shown by this team to abundantly produce chemokine CXCL4, which induced quiescence in HSCs. Another study also demonstrated association of HSCs with MKs and reported that MK ablation resulted in HSC expansion [88]. This work, however, implicated TGFβ1 in maintenance of HSC quiescence by MKs. Importantly, under chemotherapeutic stress, MK functioned oppositely to support hematopoiesis reconstitution via transient production of FGF1, which stimulated HSC expansion. A third study corroborated findings that MK depletion resulted in a loss of HSC quiescence, and implicated TPO, secreted by megakaryocytes, in maintenance of HSC [89]. The Lectin-like receptor-2 C-type (CLEC-2) protein was found to stimulate production of TPO by MKs [90]. It should be noted, however, that the role of BM-produced TPO in HSC maintenance is currently being questioned [91].

In further development of the above studies, MK depletion was found to selectively expand myeloid-biased vWF1-positive HSCs [37] As a note of caution, a recent study demonstrated that although HSCs were frequently associated with MKs, their distribution did not differ from that of randomly placed dots [36]. However, since MKs appear to control HSC proliferation through secreted proteins, direct contact between MKs and HSCs may not be necessary.

### 3.5. Macrophages

Macrophages, like other niche components, have a direct effect on HSCs in BM niches. In particular, they promote retention of HSCs in their BM niches through Nes^+^ stromal cells, as evidenced by macrophage depletion in BM that results in HSC mobilization into bloodstream [92,93]. Interestingly, VCAM-1^+^ macrophages in zebrafish have similar functions and contribute to the homing and retention of HSCs in the vascular niche through integrin A4 [94].

Another function of macrophages is to suppress the cell cycle entry of quiescent HSCs through interaction of Duffy antigen/chemokine receptor CD234/DARC on macrophages with CD82 on HSCs. This interaction activates TGF-β1/Smad signaling, whereas CD82 knockout leads to loss of HSCs due to their proliferation and differentiation [95]. In addition, a rare population of BM monocytes and macrophages with high expression of α-smooth muscle actin and cyclooxygenase COX-2 acts to suppress stress-induced exhaustion of HSCs and progenitors by production of prostaglandin E(2) and activation of CXCL12 expression [96].

It should finally be mentioned, although out of context of HSC niches, that macrophages play an exclusive and indispensable role in organization of BM erythroblastic islands, a highly specific niche for erythropoiesis containing a central specialized macrophage surrounded by differentiating erythroblasts [97]. In these structures, macrophages function to advance maturation of erythroblasts in various ways, including their mitochondrial clearance through tunnelling nanotubes [98].

### 3.6. Adipocytes

The reciprocal relationship between hematopoiesis and adipose tissue within the BM in humans has long been recognized. This notion is based on the fact that at birth bones contain red marrow essentially devoid of adipocytes and very active in hematopoiesis, while in adults red BM is replaced by yellow marrow rich in fat tissue and characterized by reduced hematopoietic activity [99]. The adipocytes, the most abundant stromal component in adult BM, were thus considered as negative regulators of hematopoiesis. This notion was experimentally supported by data indicating that in mice incapable of producing adipocytes, or after inhibition of adipogenesis by PPAR-γ receptor antagonist, hematopoiesis restoration after irradiation was significantly accelerated [100]. One of the relevant mechanisms might be a positive feedback loop in which adipocytes, via intense secretion of MCP-1, both stimulate differentiation of MSCs into new adipocytes and negatively affect HSCs [101].

Recent studies, however, have changed our perception of adipocytes as unambiguously negative HSC regulators [102]. First, adipocytes were shown to support in vitro HSC survival, proliferation, and differentiation for at least 5 weeks in culture. Importantly, in BM adipocytes production of factors with hematopoietic roles such as CXCL12, IL-8, CSF3, LIF was on par with that of MSCs [103]. Second, BM adipocytes were shown to promote regeneration of HSCs and hematopoiesis after irradiation or 5-FU treatment [104]. Adipocytes, as well as their precursors comprising a minor subpopulation of LEPR+ cells, produced SCF necessary for hematopoietic recovery. SCF from LEPR+ cells but not from endothelial or osteoblastic cells also activated regeneration, whereas conditional deletion of SCF in adipocytes inhibited hematopoietic regeneration. Thus, adipocytes formed under hematopoietic stress produce large amounts of SCF and seem to represent an emergency response providing HSCs with factors necessary for their survival and expansion [104]. Noteworthily, Wilson et al., have shown that PPARγ knockout mice lacking adipocytes exhibit severe extramedullary hematopoiesis [105]. This result might be associated with observed dysregulation of CXCL12/CXCR4 axis and suggests that adipocytes may be involved in HSC retention or mobilization.

Other reports indicate that some factors produced by adipocytes and thought to play a role in fat formation (adipokines) may be also involved in regulation of hematopoiesis. In particular, adiponectin was shown to be produced by components of the niche while its receptors are expressed by HSCs. Moreover, adiponectin increased proliferation of HSCs through a p38 MAPK-dependent pathway while maintaining their undifferentiated state, and adiponectin deficiency in mice caused negative changes in the restoration of hematopoiesis after chemotherapy [106].

Another adipocyte-secreted adipokine leptin, although alone, had little effect on survival or proliferation of mouse and human HSCs in vitro [107], synergized efficiently with SCF to stimulate the proliferation of primitive hematopoietic progenitors in vitro in colony formation by HSCs and progenitors in cultures [108]. The pleiotropy of leptin is also manifested in its effect on the differentiation of MSCs into fat cells in vivo [109].

### 3.7. Lymphoid Cells

Recent experimental evidence indicates that lymphoid cells, similar to other mature hematopoietic cells, participate in the HSCs niche function. FoxP3-positive regulatory T (Treg) cells were found to co-localize with HSCs and to play important role in protection of HSCs from immune attack in the niche. In particular, allogenic HSCs, similar to syngenic ones, are able to survive in non-irradiated mice after transplantation for at least a month, whereas depletion of Treg cells results in a rapid loss of allogenic stem cells [110]. BM niche is thus may be considered as an immunologically privileged site and a protective sanctuary for HSCs. Importantly, Treg depletion impairs function of both MSCs and HSCs and results in reduced hematopoiesis-supporting capacity of the niche [111]. Another study identified a CD150^high^ subpopulation of Treg cells in BM that through CD39 cell surface ectoenzyme produced elevated levels of extracellular adenosine. Adenosine, in turn, potentiated Tregs, protected HSCs from oxidative stress and maintained HSCs quiescence. Moreover, co-tranplantation of this Treg subpopulation was found to promote a much better engraftment of HSCs in allogenic hosts compared to other Treg subsets [112]. Paradoxically, adenosine produced by LEPR^+^ perivascular MSC seems to work in the opposite direction by activating immunity and reducing immune privilege of the niche [113].

Less evidence exists concerning the potential role of B lymphocytes in the niche. However, neurotransmitter acetylcholine abundantly produced by B cells has recently been shown to limit hematopoiesis in vivo [114].

### 3.8. Nerve Fibers

The sympathetic nervous system (SNS) has been shown to innervate BM, wrapping around arterioles and contacting peri-arterial Nes-GFP+stromal cells [32,115] A study by Méndez-Ferrer et al., demonstrated that the SNS innervation plays a key role in the circadian mobilization of HSCs form BM [116]. It acts through β3 adrenergic receptors and downregulates CXCL12 levels during daytime, which activates HSC egress from BM. Moreover, a similar mechanism functions during chronic variable stress, which induces sympathetic nerve fibers to release more noradrenaline. This in turn leads to decreased CXCL12 levels, resulting in activation of HSCs and enhanced production of neutrophils and inflammatory monocytes [117].

Importantly, the autonomic cholinergic nervous system acts in the opposite direction, repressing the sympathetic noradrenergic system at night and thus reducing egress of HSCs [118]. In addition, cholinergic system through increased CXCL12 production acts to maintain HSC quiescence in the endosteal niche under proliferative stress [119].

### 3.9. Single Cell Analysis of Niche Heterogeneity

The studies described in previous chapters demonstrated the vast diversity of BM niche cells. The explosive development of new massive parallel methods of single cell analysis has added a new dimension to our understanding of niche complexity. The majority of the relevant data has so far been obtained using single cell RNA sequencing (scRNAseq) of fluorescently sorted BM cell populations, followed by comprehensive bioinformatics analysis. The work by Wolock et al. [120] studying transcriptomes of CD45^−^, Ter119^−^, CD31^−^ BM cells used SPRING visualization algorithm [121] to reveal 7 stromal cell subsets, with MSCs sitting at the top of hierarchy and branching into adipo- and osteo/chondro-lineages. Another report by Baryawno et al. [122] analyzed substantially more cells of a broader Lin^−^, CD45^−^ BM population and employed t-distributed stochastic neighbor embedding (t-SNE) algorithm for visualization [123]. This study discovered 17 distinct subsets of non-hematopoietic cells in BM, including LEPR^+^ MSCs, endothelial sinusoidal and arteriolar cells, five fibroblast subsets, as well as several osteolineage and chondrocyte subsets. The changes in BM cells associated with emerging acute myeloid leukemia (AML) were also identified.

Tikhonova et al. [75] employed a different approach to isolate starting cell populations using transgenic mice with fluorescently marked endothelial (VE-cad^+^), perivascular (LEPR^+^) or osteo-lineage (Col1a1^+^) populations, whose transcriptomes were analyzed separately, revealing 2, 4, and 3 distinct subsets, respectively. In addition, cellular sources of haematopoietic cytokines, chemokines, and membrane-bound ligands were identified in this study. Moreover, scRNAseq analysis of 5-FU chemotherapy effects revealed drastic elevation of adipogenesis-related pathways and downregulation of osteolineage signaling during stress hematopoiesis. Zhang et al. [124] aimed to focus their analysis on BM mesenchymal cells using transgenic mice with these cells specifically marked by fluorescent protein. They identified 22 cell subsets; however, only 9 groups were of mesenchymal lineage. Authors observed a novel adipogenic lineage population expressing adipocyte markers but not containing lipid droplets. These cells form a ubiquitous 3D network in BM, maintaining marrow vasculature and suppressing bone formation.

Baccin et al. [125] used an advanced approach combining scRNAseq analysis of sorted cell subsets with spatial transcriptomics of fixed, laser capture microdissected, small (200–300 cells) samples. This work identified two new subsets of CAR cells expressing osteogenic or adipogenic gene subsets (Osteo-CAR cells or Adipo-CAR cells, respectively).

Importantly, re-analysis of results of five mentioned scRNAseq studies using the Seurat pipeline [126] for comparing all datasets demonstrated a general yet relatively limited similarity of results obtained by different groups [127], with the highest similarity observed for Baryawno et al. [122] and Tikhonova et al. [75] data. This, most likely, is a result of differences in cell subsets analyzed, as well as in cell sample digestion and processing protocols. Of note, the sc transcriptome study by Addo et al. [128] exploiting similar approaches observed a very high heterogeneity of expression phenotypes and thus was unable to identify distinct stromal cell subsets using bulk RNA abundance patterns only. However, 14 non-overlapping cell populations were identified based on the clustering of transcripts encoding secreted factors participating in stromal-hematopoietic cell communication. The reasons for this divergence from other groups’ results remain unknown.

Finally, an important study by Severe et al. [129] needs to be mentioned. Authors used a single-cell mass cytometry, a cytometry method based on the use of transition element-labeled antibodies combined with single cell mass spectrometry. This approach allows one to analyze significantly more parameters at once than is possible with conventional fluorescent cytometry. Severe et al., were able to identify 28 distinct subsets of stromal cells in BM under steady-state conditions, although only half of them expressed hematopoiesis-relevant cytokines. Importantly, radiation conditioning resulted in a loss of most of stromal cell subpopulations including LEPR^+^ and Nes^+^ niche cells. However, CD73-positive cell subset was retained following irradiation, and ablation of CD73 resulted in defects of homing of transplanted HSCs and decreased hematopoiesis. Thus, the CD73+ stromal subset is likely to promote HSPC engraftment and acute hematopoietic recovery following radiation conditioning.

Table 1 presents a brief summary of various cell types contributing to the BM niches, whereas Table 2 reviews key regulators controlling HSCs.

## 4. Niches of Committed Cells

The question arising after reviewing the specialized niches for HSCs in BM is where the potential niches for more differentiated cells are located. Ding et al., showed that while HSCs are maintained by a perivascular niche combining Lepr–cre- or Prx1–cre-expressing stromal cells and endothelium, early lymphoid precursors are located in the endosteal niche created by osteoblasts that support the proliferation and differentiation of lymphoid precursors in vitro [78]. Committed B-lineage progenitor cells are maintained by a different perivascular niche containing Prx1–cre-expressing stromal cells but no endothelial cells [78]. The removal of osteoblasts from the culture negatively affects the population of lymphoid precursors [29,86]. Moreover, the removal of G protein alpha from osteoblasts reduces the population of B lymphoid precursors [130].

The spatial distribution of hematopoietic progenitor cells is confirmed by the work of Comazzetto et al., showing that SCF from LEPR^+^ cells is generally required for HSCs and more differentiated progenitors [131]. Deletion of SCF from LEPR^+^ cells led to the depletion of common lymphoid and common myeloid progenitors as well as a number of more differentiated progenitors. At the same time, deletion of SCF from endothelial cells only negatively affected the HSC population, indicating that SCF produced by endothelial cells selectively supports HSCs.

A number of authors note that proliferating HSCs, precursors, and more differentiated cells are located in the sinusoid region [30,132]. Pinho et al., used a different cell labeling method to study the spatial distribution of cells in the niche [37] and showed that platelet- and myeloid-biased HSCs expressing von Willebrand factor (vWF) and CD150 are located near megakaryocytes, and the depletion of the latter expands the population of vWF^+^ HSCs, but reduces the ability to self-renew. At the same time, vWF lymphoid-biased HSCs in arteriolar niches are dependent on NG2^+^ cells. According to Winkler, multipotent precursors are located on the endosteal surface, whereas lymphoid precursors migrate from this region [133]. Balzano et al., demonstrated that HSCs and pro-B cells are often located in the same perisinusoidal niche next to LEPR^+^ cells [134].

## 5. Metabolic State of HSCs and Niche

Functional, transcriptomic, and proteomic studies demonstrated the importance of metabolic processes in biology of HSCs [135]. Most of the time HSCs are quiescent, which protects them against proliferative and genotoxic stress effects harmful to HSCs, while allowing them to maintain their self-renewal potential [136,137]. It is well documented that reactive oxygen species (ROS) cause damage to biomolecules, primarily DNA [138], representing a particular danger for HSCs that give rise to enormous numbers of progeny cells. Several studies established that HSCs in the BM are located in regions of severe hypoxia, with the lowest O_2_ levels in deeper peri-sinusoidal regions, while the endosteal regions are less hypoxic due to arteriole perfusion [31,139].

Quiescent HSCs in BM under these hypoxia conditions adapt their metabolism to switch to glycolysis [140] as energy source, with resulting decrease in oxidative phosphorylation (OxPhos) and production of ROS in mitochondria [136,141]. This switch is mediated by pyruvate dehydrogenase kinase (Pdk)-dependent mechanism [142]. In addition, the levels of transcription factors Meis1 and Hif-1α are highly elevated in long-term HSCs, while Meis1 positively regulates HSC glycolytic metabolism through transcriptional activation of Hif-1alpha [140]. The notion that hypoxia is necessary to maintain the functions of the HSCs is confirmed by the increasing expansion of colony-forming cells (CFCs) [143,144] or the restoration of quiescence in hypoxia ex vivo [145,146].

It should be noted, however, that ROS is an important mediator of intracellular signaling. In relation to HSCs biology, decrease in ROS below critical levels by inactivation of AKT1 and AKT2 kinases inhibits differentiation of HSCs [147]. In addition to AKT1 and AKT2, ROS levels in HSCs are regulated by ATM [148] and the Foxo transcription factor family [149,150]. The transition of HSCs to active state, including proliferation and differentiation into progenitor cells, is initiated by an increase in the concentration of ROS due to the activation of OxPhos [147,151], switching from glycolysis to the Krebs cycle to meet the rapidly growing energy needs of HSCs [135,141]. Apparently, this switch occurs through CD36-mediated uptake of free fatty acids [152] and involves histone demethylase Fbxl10 [153]. In addition, Maryanovich et al. [154] showed that loss of mitochondrial carrier homolog 2 (MTCH2) leads to an increase in OxPhos in mitochondria. This is accompanied by an increase in mitochondrial size and accumulation of ROS and ATP and, as a result, activation of HSC and an exit from the quiescent state.

The role of mitochondria is further emphasized by the finding that mitofusin 2 (Mfn2), a protein involved in mitochondrial fusion, is necessary for maintenance of lymphoid-biased but not myeloid-biased HSCs, the effect most likely mediated through intracellular accumulation of Ca^2+^ and NFAT signaling [155]. In addition, lowering the mitochondrial potential in HSCs favors their self-renewal at the expense of differentiation [156].

An interesting study demonstrates a critical function of p38MAPK family isoform p38α in transition from quiescence to proliferation [157]. After hematological stress, p38α is rapidly phosphorylated, which leads to elevated expression of IMPDH2 in HSPCs and activation of purine metabolism, whereas deletion of p38α results in suppression of recovery and delayed HSC proliferation.

Another important metabolic pathway, namely fatty acid oxidation (FAO), has also a prominent role in HSC biology. Ito et al., showed that the PML–PPAR-δ–FAO signaling controls the asymmetric division of HSCs, and its inhibition leads to symmetric divisions and loss of HSC maintenance [158]. Activation of PPAR-δ–FAO pathway leading to expansion of HSCs occurs through enhanced Parkin recruitment in mitochondria and induction of mitophagy [159]. Interestingly, depletion of PTPMT1, a PTEN-like mitochondrial phosphatase, affected mitochondrial metabolism and enhanced activation of mitochondrial uncoupling protein 2 by fatty acids, which was accompanied by block in differentiation and approximately 40-fold expansion of primitive HSC population [160].

Autophagy has also been implicated in the HSC maintenance. In particular, autophagy has a vital function in protecting HSCs from metabolic stress, and transcription factor Foxo3a is important for rapid induction of autophagy in HSCs upon starvation [161]. HSCs with suppressed autophagy accumulate mitochondria and acquire an activated metabolic state, which leads to accelerated myeloid differentiation and loss of regenerative potential, features characteristic for aged hematopoietic system. Thus, autophagy suppresses metabolism of HSCs to maintain their quiescence and stemness [162]. It should be noted, however, that excessive autophagy and mitophagy may be deleterious. For example, deletion of the mitochondrial membrane protein Atad3a (ATPase family, AAA domain containing 3A) causes hyperactivation of mitophagy, which in turn causes differentiation block with concomitant expansion of HSC pool [163].

Another work emphasizing important role of mitochondria in the HSC maintenance and function demonstrated that inactivation of histone deacetylase Sirtuin 7 (Sirt7) provokes cycling of HSCs and their decreased regenerative capacity [164]. These effects were due to suppression of mitochondrial unfolded protein response (UPRmt) in HSCs mediated by activation of nuclear respiratory factor 1 (Nrf1), a master regulator of mitochondria. Vice versa, upregulation of Sirt7 improved the functionality of aged HSCs. It has also been reported that UPRmt is activated in HSCs when they exit quiescent state [165].

Other studies demonstrated important role in HSCs biology of AMP and mTOR pathways, which sense and coordinate nutrient level, energy status and cell growth. Thus, deletion of LKB1, a well-known regulator of AMPK, was shown to reduce mitochondrial membrane potential and ATP levels, induce HSC exit from quiescence and increased proliferation resulting in rapid depletion of HSC and all hematopoietic subpopulations [166,167]. Conditional deletion of TSC1 caused activation of the mTOR signaling pathway in HSCs and provoked their entry into cell cycle, leading to the depletion of the HSC pool and, as a result, dramatically reduced hematopoiesis and self-renewal of HSCs [168].

There is a relative paucity of data regarding the metabolic state of various components of BM niches. MSCs, similar to HSCs, exist in regions of deep hypoxia in BM niches and, therefore, use glycolysis for their energy needs [169]. In the state of normoxia, the proliferative properties of MSCs increase, but aging also increases [170], therefore hypoxia might serve as a way to prevent proliferation and aging of MSCs, maintaining self-renewing MSC population of BM.

Finally, in the context of mitochondria role in HSC function, it would be important to discuss the issue of mitochondrial transfer between cells in BM. According to numerous reports, MSCs, a vital component of HSC niches, are able to donate their mitochondria to various cell types in vitro, either through direct contact or via secretion of extracellular vesicles (reviewed in [171]). This process modulates properties of recipient cells and, frequently, results in restoring their functions. Moreover, MSCs may sense mitochondria released from injured cells and enhance their own mitochondrial biogenesis to combat cell injury [172]. It is thus possible that a similar functional restoration of HSCs containing damaged or exhausted mitochrondria by niche MSCs may occur in vivo. Of note, a reverse process of mitochondrial donation by healthy hematopoietic progenitor cells to damaged irradiated stromal cells during bone marrow transplantation has been reported [173].

## 6. Aging of the Niche

Age-related changes in the human body negatively affect hematopoiesis, leading to shifts in blood cell clonal composition, DNA damage and eventual development of hematological disorders [174,175,176]. The aging of the hematopoietic system involves two major components: the cell-intrinsic aging of the HSCs themselves, and age-related changes in the components of the BM niche that have a direct impact on the HSCs and hematopoiesis.

Aging affects HSCs in two ways: on the one hand, HSC functional capacity declines with accumulation of genetic, epigenetic, metabolic and homeostatic defects, on the other hand, the HSC pool expands, with a skewed differentiation towards the myeloid lineage at the expense of the lymphoid one [176,177,178]. Age-related changes are also associated with DNA and telomere damage; for aged HSCs, an increased level of DNA mutations was observed [179]. Telomere shortening leads to functional depletion of HSCs due to constitutive activation and asymmetric differentiation into megakaryocytes [180]. Accumulation of mutations in epigenetic regulator genes in HSCs with age leads to clonal hematopoiesis, enhanced inflammation and increased risk of cardiovascular diseases and malignancies [181,182]. The aging of HSCs is manifested at the organelle level as well. Various defects in the mitochondrial respiratory chain or mitochondrial biogenesis lead to disturbance of HSC quiescence and their dysfunction [164,168,183]. Among other prominent hallmarks of HSC aging are deregulation of autophagy [162,184] and modifications in epigenome [185,186]. Importantly, HSCs lose polarity with aging as a result of enhanced activity of the small RhoGTPase Cdc42 [187]. It should be noted that the biologically youngest HSC subset in the aged mice that retains cellular polarity, quiescence, and conserved regenerative potential was shown to be located in perisinusoidal niches [188].

HSCs do not age alone in BM; age-related changes occur in the BM niche components as well. In a number of adult stem cells, aging is accompanied by a continuous somatic DNA mutagenesis occurring at a rate of about 40 novel mutations per year [189]; the same is likely to occur in MSCs as well. The aging of MSCs is accompanied by an increase in ROS levels and DNA damage [190,191,192]. The lack of telomerase negatively impairs the ability of MSCs to differentiate [193]. Moreover, telomere dysfunction in Tert knockout mice enhanced myelopoiesis during aging at the expense of B lymphoid lineage, impaired MSC function and reduced their capacity to maintain functional HSCs, in particular their ability to support the engraftment of wild-type HSCs in KO animals [194].

The secretory ability and immunomodulatory properties of MSCs significantly weaken during aging in culture [195]. In line with in vitro data, Gnani et al., showed that MSCs isolated from aged donors displayed senescent phenotype, accumulation of DNA damage and increased secretion of multiple pro-inflammatory factors with concomitant reduction in immunomodulatory properties [196]. Factors secreted by aged MSCs activated expression of proinflammatory genes in young HSCs and reduced their clonogenic potential.

The vascular component also undergoes major alterations in the advanced age. Although the vascular volume remains fairly constant, a decrease in the number of ECs occurs [188]. With age, degeneration of arteries and arterioles occurs [197], they become disorganized, which impairs their ability to maintain HSC quiescence and affects function of the latter [188,198]. At the same, sinusoidal network undergoes little degradation [198]. Netrin-1 expressed by endothelial and periarteriolar stromal cells of the BM niche was recently reported to support HSC quiescence and self-renewal, while decline of its production with ageing negatively affects HSC maintenance [199]. It is worth noting that other genes in ECs have also been implicated in the aging of the niche. In particular, expression of mTOR and heme oxygenase-1 in BM ECs declines during aging, which impairs support of hematopoiesis [200,201].

Substantial alterations occur in the endosteal compartment with aging. The ability of MSCs to differentiate into osteoblasts decreases with aging, which is associated with decreased secretion of osteopontin, which, in turn, negatively affects the population of HSCs [83]. In aged mice, the bone matrix is reduced, and the number of mature osteoblasts diminished [202]. A decrease in the number of osteocytes in the niche leads to a shift in the differentiation of HSC towards myelopoiesis [203], which is further stimulated by the influx of plasma cells during aging [204].

The adipose component also changes with aging. Shift in MSCs during aging towards differentiation into adipose tissue results in niche remodeling associated with pathological accumulation of adipocytes, which disrupts hematopoietic regeneration [205]. It is assumed that accumulation of adipocytes in the niche can affect the immunomodulatory activity of MSCs, increasing or weakening the inflammatory state and contributing to a decrease in the population of HSCs [205,206,207].

In the aging BM, a decrease in sympathetic adrenergic nerve density and innervation was reported, with resulting increase in HSCs proliferation, loss of Cdc42 polarity, myeloid shift in differentiation and decrease in HCS transplantation potential [197]. However, a more recent study, on the contrary, reported an increase in sympathetic innervation with age, which promoted myeloid differentiation and increased HSC frequency [208].

One of the important negative changes in aging is development of the inflammatory process (inflammaging) in the niche [209]. Activation of signals associated with aging, such as NF-kB, β-catenin/WNT, elicits a pro-inflammatory shift in MSCs that provokes the expression and secretion of senescence-associated pro-inflammatory cytokines, chemokines and ligands, such as S100A8/A9, IL6, IL-1ß and others, into the niche [210]. Such a proinflammatory environment in the niche favors DNA damage, malignant transformation and the appearance of mutations in HSCs, which may lead to oncogenesis [196,211,212,213].

Finally, a number of interesting aging-related experiments were performed to elucidate the heterochronic interactions of HSCs with the hematopoietic niche. Transplantation of aged HSCs into young mice did not result in their conversion to a younger state and functionality, although a rejuvenation of HSC transcriptome, but not methylome, was observed [214,215]. Similarly, aged microenvironment had little pro-aging effect on transplanted young HSCs [214], although young HSC pool expansion biased towards myeloid differentiation was reported [216,217]. These data indicate that HSCs largely maintain their chronological age in the heterochronic environment.

Table 3 summarizes changes in HSC-niche system that occur during aging.

## 7. Niche Transformation in Leukemia

Numerous data indicate that during leukemia, malignant cells, in addition to perturbation of normal hematopoiesis, are able to substantially modify the BM niche components, and the hematopoietic microenvironment undergoes changes that facilitate disease progression. In particular, during chronic lymphocytic leukemia BM MSCs change their properties to give rise to drastically fewer fibroblast colony-forming units (CFU-Fs), proliferate slowly and behave as senescent cells [218]. Senescence was also observed for MSCs from AML BM [219]. Moreover, MSC senescence is reported for an in vitro leukemia niche model [220].

One of the leukemia hallmarks is enhanced angiogenesis, which is activated by proangiogenic factors secreted by tumor cells. This “superangiogenesis” allows rapidly proliferating malignant cells to spread throughout the body and is also an indicator of leukemia progress [221]. The key angiogenesis factor VEGF activates protection of leukemic cells from apoptosis by Hsp90-medited induction of anti-apoptotic factors BCL2 and MCL1 [222,223]. In co-cultures of endothelial cells with AML, an increase in secretion of IL-3, IL-6, G-CSF and GM-CS cytokines was observed, which stimulated AML growth and suppressed apoptosis [224].

In connection to the rapid proliferation of leukemic cells in BM, adipocytes acquire small size due to lipolysis [225]. As a result, a large amount of free fatty acids is formed, which are necessary to maintain the existence of malignant cells [226]. Transition from larger to small adipocytes during AML [227] was mediated by growth differentiation factor-15 (GDF15) secreted by leukemic cells, which enhanced expression of thermogenic and lipolytic genes stimulating lipolysis [228]. Notably, disruption of the adipocyte niche in leukemia BM negatively affects endogenous myelo-erythropoiesis [229]. Among other niche components, AML was shown to affect sympathetic nervous system in BM. The resulting neuropathy affects the Nes^+^ MSCs that support quiescent HSCs in the niche, inducing MSC differentiation into precursors primed for osteoblastic differentiation [230].

The BM microenvironment plays a substantial role in leukemia progression. Leukemia clones at first propagate locally in BM and then spread to other regions in bones. In particular, leukemia cells home to BM vasculature expressing CXCL12 and E-selectin [231]. CXCL12 deletion from vascular endothelial, but not perivascular, cells impeded T-cell acute lymphoblastic leukemia (T-ALL) [232]. Similar effects were reported for deletion of the CXCR4 (CXCL12 receptor) in T-ALL cells.

In the context of leukemia pathology, it would be important to mention that normal HSCs placed in the leukemia niche in vitro activate the cell cycle and start to intensely proliferate [220]. One of the major mechanisms responsible for this effect is the exosome-mediated intercellular communication between malignant and normal cells [233,234]. Leukemic cells capture and endocytose MSC-produced exosomes containing fibroblast growth factor 2 (FGF2), which protects against tyrosine kinase inhibitors [235]. Moreover, leukemic exosomes are able to stimulate the production of IL-8 by BM MSCs, which in turn supports the spread of leukemia [236]. The ability of leukemic cells to remodel the normal niches manifests in the fact that exosomes from these cells are able to enter and significantly modify the surrounding cells. By virtue of their RNA and protein load, exosomes modulate the secreting profiles of niche cells, accelerating leukemic growth and advancing leukemia progress [237,238,239]. Yet another mechanism was observed in the study by Marlein et al. [240], where production of superoxide by NADPH oxidase-2 in AML stimulated transfer of mitochondria from BM MSCs to AML cells through tunneling nanotubes.

It should be noted that the pathological remodeling of the niche by leukemia cells is not the only way of how the BM microenvironment may promote hematological disorders. Since the niche is thought to strictly control proliferation of HSCs and progenitors, genetic lesions of niche cells may have a disturbing effect on hematopoiesis. Thus, mice deficient in retinoic acid receptor gamma (RARγ) develop myeloproliferative syndrome with abnormal myeloid specification and proliferation, which is elicited solely by the defects in microenvironment [241]. Inactivation of Mind bomb-1, which participates in endocytosis of ligands of Notch receptor, results in myeloproliferative disease with accumulation of immature granulocytes that was due to abnormally functioning non-hematopietic BM cells [242]. Moreover, activating β-catenin mutation in osteoblasts results in upregulation of Notch ligand Jagged 1, which leads to leukemogenesis [243].

Finally, it would be important to discuss the significance of leukemic niche studies for development of new therapeutic strategies. Although the current anti-leukemia therapies target exclusively the malignant cells proper, the accumulating evidence for active and vital synergism between leukemic niche and leukemia cells suggests that this synergism might be a novel and promising therapeutic target. This field is still at its beginning, however, and it should be mentioned that the initial attempts to target vascular niche using anti-angiogenic drugs have been largely unsuccessful [244]. At the same time, targeting niche-leukemia interaction seems to hold more promise. In particular, blocking interaction of CXCR4 with its ligand CXCL12 by plerixafor demonstrated efficacy in several combination trials in AML [245,246,247]. Currently, a number of other substances blocking the CXCL12-CXCR4 axis, such as BL 8040, CX-01 and ulocuplumab, are in early phase trials [248,249,250].

Inhibition of E-selectin in vascular niche by uproleselan resulted in a promising outcome in a phase 1/2 clinical trial of relapsed/refractory AML [251]. Among the most promising targets is also an E-selectin ligand CD44, in particular its CD44v6 isoform expressed in AML SCs but not in normal HSCs. For detailed information on translational research in the field of leukemia-niche interactions, the reader is referred to recent excellent reviews [245,252,253].

## 8. Niche Modeling

HSC transplantation is one of the most powerful tools for the treatment of blood diseases, resulting in the restoration of functioning hematopoiesis in the body. However, HSC transplantation is limited by the availability of suitable HLA-matched donors and risks associated with GVHD development, insufficient graft quantity or its rejection [254]. These problems could be solved by developing an ex vivo system for expansion of donor HSCs in the quantities required for successful transplantation.

The developers of these ex vivo HSC expansion systems are facing a formidable problem of mimicking the complexity of the hematopoietic niche combined with a correct blend of external signals. To create a dynamic niche model, a combination of cells, growth factors/cytokine and sufficiently elastic biomaterials providing a 3D structure is being used [255,256]. The key supporting elements in making niche lookalikes are arguably the 3D scaffolds [257]. The ex vivo system should be functionally, if not structurally, similar to BM in order to ensure long-term HSC well-being and expansion. For the creation of 3D scaffolds, synthetic, natural and biomaterials are used that may provide a suitable framework for attachment, survival and free movement of cells.

The problem of biocompatibility sets certain restrictions on the choice of materials for 3D structures [258]. Hydrogels are among the most biocompatible materials and are being widely used to prepare scaffolds imitating BM structures [256,259,260,261]. Thus, Ravichandran et al., developed a model of BM adipose tissue by combining gelatin and a composite methacryloyl hydrogel/polycaprolactone scaffold with human BM MSCs subjected to mechanical stimulation [262]. Scaffolds from polydimethylsiloxane used as BM mimics allowed for the successful cultivation of HSCs, which, in the 3D culture, activated pathways maintaining cell pluripotency and promoting their expansion [263]. Studies by Ventura Ferreira et al., using co-cultures of cord blood CD34^+^ cells and MSCs on various 3D biomaterial scaffolds revealed that the fibrin-based scaffold provided best culture conditions and maximally contributed to the expansion of CD34^+^ cells [264]. The use of biocompatible zwitterion hydrogels made it possible to improve the properties of the scaffolds and achieve a multifold increase in the frequency of hematopoietic progenitor cells capable of long-term hematopoietic reconstitution in immunocompromised mice [265]. Other types of scaffolds are also being used to recreate the niche, and their low biocompatibility is improved by coating nanofibers with fibronectin [266,267], collagen [268] or by prior cultivation of MSCs [268]. A ceramic scaffold pre-populated with MSCs and osteoblasts, which secreted ECM and cytokines necessary for the maintenance of hematopoietic progenitors, successfully promoted the expansion of the latter [269].

Born et al., created a system imitating BM using the stromal vascular fraction (SVF) from human adipose tissue seeded on 3D osteoblastic niches formed by MSCs, in which SVF cells assembled into vascularized structures containing both endothelial and perivascular cells, and which provided a better support for HSCs than the non-vascularized 3D niches [270]. An artificial niche created from silk fibroin reproduced platelet biogenesis [271]. The use of natural materials—collagen and fibrin—in the design of the scaffold made it possible to support the proliferation and expansion of HSCs [259,264].

Another strategy for creation of biomimetic 3D scaffolds is the use of decellularized animal and human tissue. This enables one to simulate the architecture of the niche for cell cultivation as close to the natural environment as possible while eliciting a smaller host immune response during transplantation [272,273]. Properly performed decellularization of the tissue diminishes an unfavorable immune response [274,275]. Bianco et al., created a novel decellularized BM scaffold using a cell removal method that preserves the native 3D-structure of BM with its blood vessels and niches, thus supporting adhesion and proliferation of both stromal cells and HSCs [276]. Hashimoto et al., employed high hydrostatic pressure for bone decellularization, which allowed HSC recruitment after subcutaneous transplantation [277]. Lai et al., used yet another option for co-cultivation of HSCs and MSCs, namely, the decellularized bone-like matrix containing osteogenically differentiated MSCs encapsulated in collagen microspheres [278].

One of the options for modeling the BM niche is the cultivation of HSCs, MSCs and ECs in 3D multicellular spheroids using a magnetic levitation system [279]. This study demonstrated, in general agreement with previous data, that exposure of spheroids to hypoxia is beneficial for HSCs, resulting in enhanced cell proliferation and increased expression of CD34 and some other markers. Spheroid culture (hematosphere) from mononuclear blood cells demonstrated the possibility of maintaining and expansion of Lin^−^CD34^+^CD38^−^ hematopoietic progenitors [280]. The creation of vascularized miniature bone/BM organoids in mice using umbilical cord blood and cord blood fibroblasts allowed for successful engraftment and maintenance of HSCs [281].

A highly promising way to create and monitor the niche as a constantly changing microenvironment is the organ-on-a-chip technology based on microfluidics. With the help of porous membranes or scaffolds and a system of microchannels, a suitable environment is created in which MSCs are seeded to prepare an appropriate niche for HSCs. Kefallinou et al., created such an organ-on-a-chip system using two chambers separated by a porous membrane, in one of which MSCs were cultivated [282]. In another application of organ-on-a-chip technology, a zirconium oxide scaffold coated with hydroxyapatite was used to co-cultivate MSCs and hematopoietic progenitors in the microfluidic chip [283]. This system created a microenvironment similar to BM that supported maintenance of CD34^+^ CD38^−^ progenitors. Glaser et al., presented a microfluidic 3D model of BM combining two perivascular and endosteal compartments and a perfusable vascular network, which successfully maintained CD34^+^ cells and allowed for their differentiation and egress of neutrophils [284]. For a detailed study of multiple myeloma, a microfluidic system imitating BM stromal and sinusoidal endothelium components, as well as sinusoidal circulation, made it possible to study CXCL12-mediated egress of myeloma cells from BM stroma [285]. The use of microfluidic technology for niche modeling will undoubtedly undergo further development. For example, the application of the method of maskless photolithography to create more sophisticated microfluidic devices that can be adapted for specific research needs holds significant promise in further progress of organ-on-a-chip systems [286].

As a final note, it would be important to mention the 3D printing technology, a potentially very powerful approach considered one of the major avenues for creation of artificial tissues and organs, despite tremendous technical and methodological hurdles that are lying ahead [287,288]. In the field of stem cell biology, this technology might eventually allow one to re-create sophisticated nature-like stem cell niches using biomaterials and various cell types [289]. Although this field is still in its infancy and no major breakthroughs have been yet reported, some steps towards achieving the above goal have already been made [290,291].

## 9. Concluding Remarks

The hematopoietic system is arguably unique among the body’s systems in the numeric cellular output and variety of cell types it generates. The sheer number of cells it produces daily presents a significant potential danger of malignant transformation or inability to correctly meet the various needs of organisms. Given this, it is no wonder that hematopoiesis is subjected to a very strict control, while HSCs are kept on a short leash and their behavior is tightly regulated by multiple neighbor cells that constitute the niche for HSCs. Unraveling the enigmas of different molecular and cellular mechanisms underlying HSC-niche interactions can thus be considered the Holy Grail of hematopoietic research. The most sophisticated techniques such as genetic lineage tracing and single cell transcriptome analysis are being increasingly used to understand the astonishing complexity of hematopoietic regulation by the BM microenvironment. Experiments performed until now have already yielded quite a few important discoveries in this field that are changing our paradigms. However, the unified and commonly accepted view of how HSCs interact with the BM microenvironment to fulfill their vital functions is still lacking. Although at least some of the major HSCs regulators produced by niche cells have been identified, we are as yet very far from obtaining an integral picture of how all numerous actors of the HSC-niche ecosystem work together to help HSCs to make correct decisions concerning their survival, division and differentiation in response to organism’s needs.

Future research will undoubtedly focus of these issues, and one may hope that it will not only advance our understanding of the complexities of HSC regulation by the niche, but also provide vital clues as to the identity of molecules and interactions that are required to maintain and multiply HSCs for clinical applications, as well as combat hematopoietic diseases.

## Figures and Tables

**Table 1:** Key cell types participating in regulation of HSCs activity in BM niches.

Cells	Markers	Function	Main Molecules
**Mesenchymal stem cells**	CD73+, CD90+, CD105+, CD14−, CD19−, CD34−, CD45−, HLA−DR−, Terl119−, CD31−, CD51+, PDGFRa+, Sca1−	Support and regulation of HSC quiescence, proliferation, differentiation HSC mobilization	CXCL12, SCF, angiopoietin-1, VCAM-1, osteopontin
**Endothelial cells**	CD45−, CD31+, CD144+, Terl119−	Support of HSC proliferation and expansion Hematopoietic regeneration after irradiation	CXCL12, SCF, Notch ligands, E-selectin, Del-1, pleiotrophin
**Osteoblasts**	CD45−, Terl19−, CD31−, CD51+, PDGFRa−, Sca1−	Support of HSC quiescence	Osteopontin, N-cadherin, TPO, angiopoietin-1
**Megakaryocytes**	CD41+, CXCR4, Mpl	Support of HSC quiescence HSC expansion after irradiation	CXCL4, TGF-β, thrombopoietin, FGF1
**Macrophages**	CD68+, CD169+	HSC retention in niche Support of HSC quiescence	VCAM-1, DARC, TGF-β
**Adipocytes**	ADIPOQ, FABP4, Leptin	Support of HSC survival, proliferation and differentiation Hematopoietic regeneration after irradiation	MCP-1, CXCL12, SCF, IL-8, LIF, CSF3, adiponectin, leptin
**Treg lymphocytes**	FOXP3	Protection of HSC from immune attack Support of HSC quiescence	IL-10, CDC39, adenosine
**Sympathetic nerve fibers**	Tyrosine hydroxylase	HSCs mobilization	Noradrenaline
**Parasympathetic nerve fibers**	Choline acetyltransferase	HSC retention in niche, homing	Acetylcholine

**Table 2:** Key molecules participating in HSC regulation by BM niches.

Molecule	Receptors on HSC Surface	Producing Cells	Regulatory Function
CXCL12	CXCR4	MSCs, ECs, ADs	HSC maintenance HSC homing and niche retention
SCF	KIT	MSCs, ECs, ADs	HSC survival HSC maintenance and proliferation HSC homing
TGF-β	TGFBR1	MKs	HSC quiescence
Osteopontin	CD44	OBs, MSCs	Suppression of HSC proliferation and expansion
Angiopoietin 1	TIE2	OBs, MSCs	HSC maintenance HSC quiescence
VCAM-1	VLA4	MSCs, Mϕ	HSC homing
G-CSF/CSF3	CSF3R	MSCs	Myeloid differentiation HSC mobilization
M-CSF/CSF1	CSF1R	MSCs	Myeloid differentiation
TPO	MPL	MKs, OBs	HSC maintenance and proliferation HSC quiescence
IGF-1	IGF1R	OBs	HSC maintenance
Pleiotrophin	RPTPZ1	ECs, MSCs	HSC maintenance
Jagged-1	NOTCH	MSCs, ECs, OBs	HSC maintenance and self-renewal HSC expansion
EGF	EGFR	ECs	HSCs survival and maintenance
DARC	CD82	Mϕ	HSC quiescence
CXCL4	CXCR3B	MKs	HSC quiescence

HSCs—hematopoietic stem cells, MSCs—mesenchymal stem/stromal cells, ECs—endothelial cells, ADs—adipocytes, MKs—megakaryocytes, OBs—osteoblasts, Mϕ—macrophages.

**Table 3:** Phenotypic and functional aging-related alterations in HSCs and BM niche cells.

Cell Types	Changes with Ageing
**HSCs**	↑myeloid differentiation; ↓lymphoid differentiation; ↓regenerative potential; ↓HSC polarity; ↓autophagy; ↑deregulated mitochondrial activity; ↑epigenetic and genomic alterations
** MSCs **	↓CFU-F clonogenicity; ↓*Nes*–GFP+ and NG2+ cells; ↓CXCR4 →↑ROS production ↑DNA damage→↓ HSCs support; ↑IL6 expression, ↑TGF-β expression →aged HSCs phenotype
**ECs**	↓ECs number, vascular remodeling → loss of HSC quiescence; ↓key signaling pathways in ECs (mTOR, Jag1/Notch, CXCL12, SCF); ↓HO-1 expression →aged HSC phenotype
**OBs**	↓ OBs number →↓OPN secretion → aged HSC phenotype; ↓osteogenic progenitor population
**MKs**	↑ MKs number
**Mϕ**	↑ Mϕ number; ↑IL-1 secretion→↑HSC myeloid differentiation
** ADs **	↑ADs number →↓HSCs and progenitors numbers→↓repopulation capacity
**Nerve fibers**	↓nerve density; ↑β2-adrenergic stimulation ↑→IL6 secretion by MSCs →↑HSC myeloid differentiation

↓—decrease, ↑—increase, →—leads to, HSCs—hematopoietic stem cells, MSCs—mesenchymal stem/stromal cells, ECs—endothelial cells, ADs—adipocytes, MKs—megakaryocytes, OBs—osteoblasts, Mϕ—macrophages.
